# Plant homocysteine, a methionine precursor and plant’s hallmark of metabolic disorders

**DOI:** 10.3389/fpls.2022.1044944

**Published:** 2022-12-08

**Authors:** Ewa Sobieszczuk-Nowicka, Magdalena Arasimowicz-Jelonek, Umesh Kumar Tanwar, Jolanta Floryszak-Wieczorek

**Affiliations:** ^1^ Department of Plant Physiology, Faculty of Biology, Adam Mickiewicz University, Poznań, Poland; ^2^ Department of Plant Ecophysiology, Faculty of Biology, Adam Mickiewicz University, Poznań, Poland; ^3^ Department of Plant Physiology, Poznań University of Life Sciences, Poznań, Poland

**Keywords:** homocysteine, non-proteinogenic amino acid, homocysteine derivatives, methionine metabolism, stress biomarker(s)

## Abstract

Homocysteine (Hcy) is a sulfur-containing non-proteinogenic amino acid, which arises from redox-sensitive methionine metabolism. In plants, Hcy synthesis involves both cystathionine β-lyase and *S*-adenosylhomocysteine hydrolase activities. Thus, Hcy itself is crucial for *de novo* methionine synthesis and *S*-adenosylmethionine recycling, influencing the formation of ethylene, polyamines, and nicotianamine. Research on mammalian cells has shown biotoxicity of this amino acid, as Hcy accumulation triggers oxidative stress and the associated lipid peroxidation process. In addition, the presence of highly reactive groups induces Hcy and Hcy derivatives to modify proteins by changing their structure and function. Currently, Hcy is recognized as a critical, independent hallmark of many degenerative metabolic diseases. Research results indicate that an enhanced Hcy level is also toxic to yeast and bacteria cells. In contrast, in the case of plants the metabolic status of Hcy remains poorly examined and understood. However, the presence of the toxic Hcy metabolites and Hcy over-accumulation during the development of an infectious disease seem to suggest harmful effects of this amino acid also in plant cells. The review highlights potential implications of Hcy metabolism in plant physiological disorders caused by environmental stresses. Moreover, recent research advances emphasize that recognizing the Hcy mode of action in various plant systems facilitates verification of the potential status of Hcy metabolites as bioindicators of metabolism disorders and thus may constitute an element of broadly understood biomonitoring.

## Introduction

Homocysteine (Hcy) was first synthesized by Butz and de Vigneaud in 1932 as homocystine (disulfide) by chemical demethylation of methionine (Met) with sulfuric acid. Then, three years later Hcy was obtained by reduction of homocystine with metallic sodium-ammonia (Riegel and de Vigneaud, 1935). Although Hcy is a naturally occurring intermediate in the metabolism of Met and cysteine (Cys), its excessive production might be harmful to various human and animal cells (Roe et al., 2002; Tuite et al., 2005; Jakubowski, 2006; Kumar et al., 2006; Zimny et al., 2006; Sikora and Jakubowski, 2009). Higher concentrations of Hcy were shown to affect growth inhibition or reduce cell viability in *Escherichia coli* cultures and yeast cells (Tuite et al., 2005; Sikora and Jakubowski, 2009). In animal cells an excessive level of Hcy is associated with a risk of various disorders, such as neurodegenerative and cardiovascular diseases, diabetic retinopathy, embryo developmental anomalies, certain neoplasms, and osteoporosis (Jakubowski, 2006; Smith and Refsum, 2021). Recent years have shown a dramatic increase in research aimed at providing better understanding of this exciting amino acid in animal organisms. However, still little is known concerning the role of Hcy in plant systems, in which it is only perceived as an intermediate product of Met biosynthesis and a by-product of *S*-adenosylmethionine (AdoMet) metabolism.

## Mechanisms of Hcy toxicity in living cells

The harmful effects are exerted not only by Hcy, but also by the more reactive Hcy-related metabolites emerging due to elevated levels of this amino acid in the cellular environment (Škovierová et al., 2016). As a thiol Hcy can autoxidize, while in the presence of transition metals it promotes reactive oxygen species (ROS) formation (Hogg, 1999). *In vitro*, Hcy was proven to act as a pro-oxidant through hydrogen peroxide production during metal-catalyzed oxidation (Perna et al., 2003a). Thus, an excess of Hcy may trigger uncontrolled oxidation product formation in the cellular environment, resulting in oxidative stress (Perna et al., 2003b). Wu et al. (2019) summarized that Hcy can also induce ROS production by NADPH oxidases and endothelial nitric oxide synthase uncoupling. Finally, Hcy-induced oxidative stress may arise from targeting enzymatic antioxidants including glutathione peroxidase and protein disulfide isomerase (Jacobsen et al., 2005).

Jakubowski (2006) proved that the pathophysiological mode of action of Hcy is closely related to the “Hcy-thiolactone hypothesis”. The Hcy metabolite, synthesized by methionyl-tRNA synthetase in an error-editing reaction, is a cyclic thioester with a unique repertoire of chemical reactions (Ganapathy et al., 2009; Jakubowski, 2019). As demonstrated in the *Saccharomyces cerevisiae* model yeast, both cytoplasmic and mitochondrial methionyl-tRNA synthetases are engaged in the biosynthesis of the Hcy metabolite (Senger et al., 2001). The formation of Hcy-thiolactone requires ATP and thus causes nonproductive consumption of cellular energy. Once formed, Hcy-thiolactone is relatively stable, as its half-life has been estimated to be approximately 25 h under physiological pH (pH 7.4) and temperature (36°C) (Jakubowski and Guranowski, 2003).

The total production of Hcy-thiolactone depends on the concentration of free Hcy and the Met-Hcy ratio. Notably, the unique Hcy derivative can quickly diffuse through cellular membranes owing to its amino group’s relatively low pKa value (~ 7.2) (Jakubowski and Guranowski, 2003; Chubarov, 2021). Thus, Hcy-thiolactone was also detected in extracellular media (Jakubowski et al., 2000). Hcy-thiolactone is much more toxic than Hcy and can trigger apoptosis even at low concentrations (Chubarov, 2021). In addition, it may have an inhibitory effect on Na^+^/K^+^-ATP-ase, altering the membrane potential with a deleterious effect on cells (Rasić-Marković et al., 2009).

The process of Hcy or Hcy-thiolactone incorporation into proteins is known as homocysteinylation. It can involve *S*-homocysteinylation (*S*-Hcy-protein) or *N*-homocysteinylation (*N*-Hcy-protein). As Hcy-thiolactone can only arise from Hcy, *N*-homocysteinylation constitutes a unique post-translational protein modification for Hcy (Jakubowski, 2019). *N*-homocysteinylation involves the modification of protein lysine residues and can alter or impair the protein structure and function, resulting in protein damage. The phenomenon of protein *N*-homocysteinylation is irreversible, while the accumulation of *N*-Hcy-proteins could promote proinflammatory, prothrombotic, and proatherogenic properties, contributing to various disorders associated with hyperhomocysteinemia in humans (Jakubowski, 2006). In addition, post-translational protein modification can influence the epigenetic regulation of gene expression, preventing histone lysine methylation and acetylation (Wada et al., 2018). It is important to note that *N*-homocysteinylation may also influence the susceptibility of a protein to proteolysis, as confirmed in the case of *N*-Hcy-albumin (Glowacki and Jakubowski, 2004). Experimental evidence shows that *N*-Hcy-protein formation is a phenomenon shared by various multicellular organisms, including plants (Jakubowski and Guranowski, 2003). In the case of *S*-homocysteinylation Hcy is linked *via* a disulfide bond to a free protein sulfhydryl residue. The post-translational modification may change the function of proteins *via* the inactivation of potentially active free thiol groups and shifting the redox potential of biomolecules (Chubarov, 2021). Compared to *N*-homocysteinylation mediated *via* Hcy-thiolactone, protein *S*-homocysteinylation is reversible and is not specific to Hcy, as other low-molecular-weight thiols may form disulfide bonds with protein sulphydryl residues (Jakubowski, 2019).

In the presence of the signaling molecule, nitric oxide (NO), Hcy can undergo S-nitrosation to *S*-nitroso-Hcy. As underlined by Morakinyo et al. (2010), the NO-dependent modification of the thiol group in Hcy can constitute a prevention mechanism against the metabolic conversion of Hcy to more toxic Hcy-thiolactone. In plants, NO-dependent post-translational modifications of SAHH and other crucial components of the S-adenosylmethionine cycle can also affect the Hcy level and consequently, the DNA methylation status (Lindermayr et al., 2005; Chaki et al., 2009; Lozano-Juste et al., 2011; Puyaubert et al., 2014; Hu J. et al., 2015).

## Hcy formation in plants

**Figure 1:**
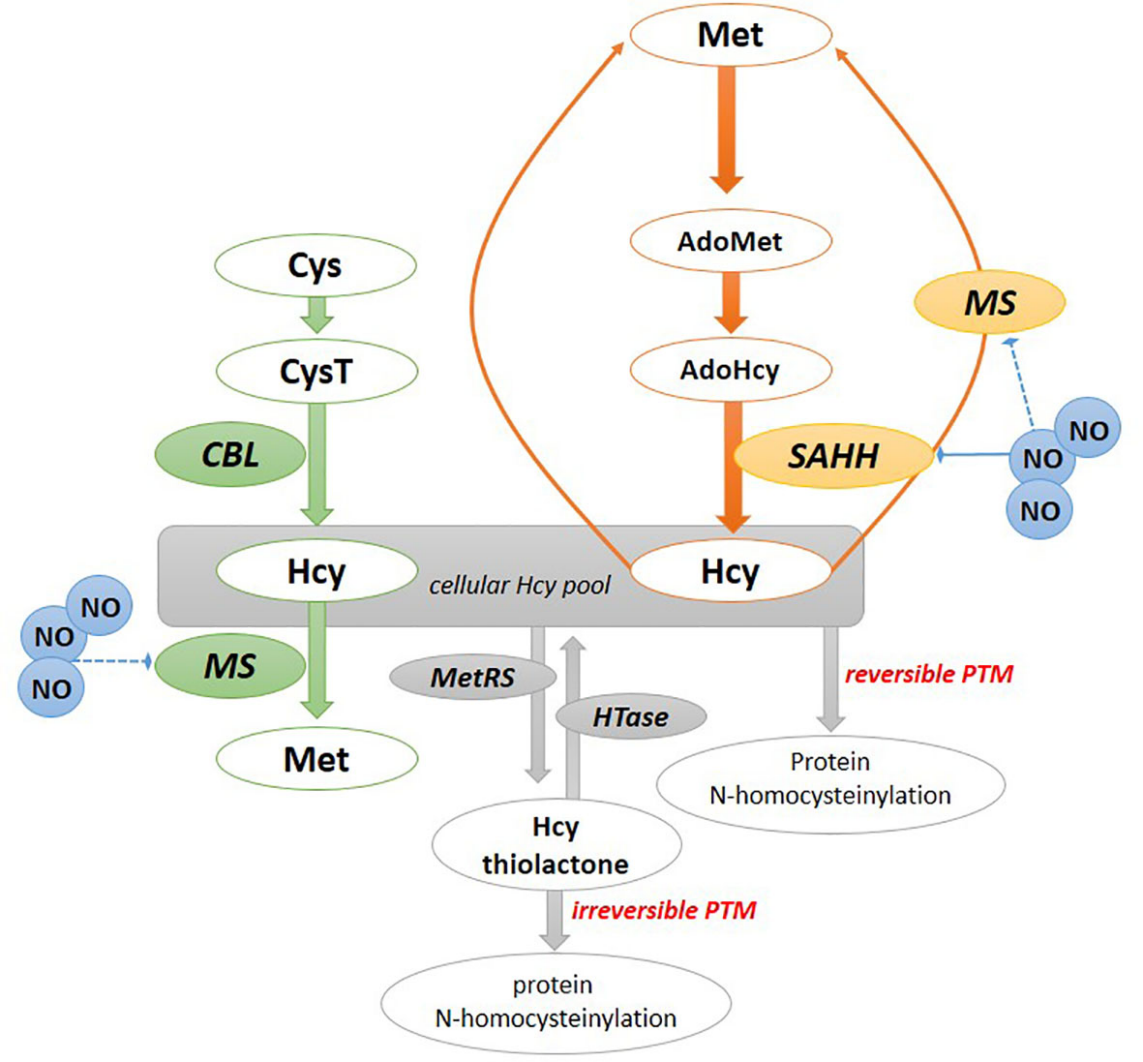
Homocysteine formation and mode of action in plants. The chloroplast pathway of Hcy formation (in green); the cytosolic pathway of Hcy formation (in orange). Hcy can be converted to Hcy-thiolactone, which modifies proteins by *N*-homocyteinylation. Hcy can also bind to cysteine residues of a protein, forming a disulfide bound resulting in *S*-homocysteinylation of proteins. AdoHcy, *S*-adenosylhomocysteine; AdoMet, S-adenosylmethionine; Cys, cysteine; CysT, cystathionine; CBL, cystathionine β-lyase; Hcy, homocysteine, HTase, Hcy-thiolactone hydrolase; MetRS, methionyl-tRNA synthetase; MS, methionine synthase; NO, nitric oxide; SAHH, *S*-adenosylhomocysteine hydrolase.

As an immediate methionine precursor, Hcy is synthesized in plant cells *via* two pathways (**Figure 1**). One of them involves the plastid/chloroplast and includes the route from sulfate *via* cysteine and cystathionine (CysT) formation; however, next to cysteine also O-phosphohomoserine can be metabolized to CysT by CysT γ-synthase. Finally, b-cleavage of CysT to Hcy is catalyzed by cystathionine β-lyase (CBL) (Ravanel et al., 1998; Hesse et al., 2004; Ravanel et al., 2004). The other cytosol route involves Hcy formation as a by-product of the methylation reaction within plant cells (Jakubowski and Guranowski, 2003). In this respect, *S*-adenosylhomocysteine (AdoHcy) undergoes conversion into Hcy in a reaction catalyzed by *S*-adenosylhomocysteine hydrolase (SAHH) (Ravanel et al., 2004).

**Figure 2:**
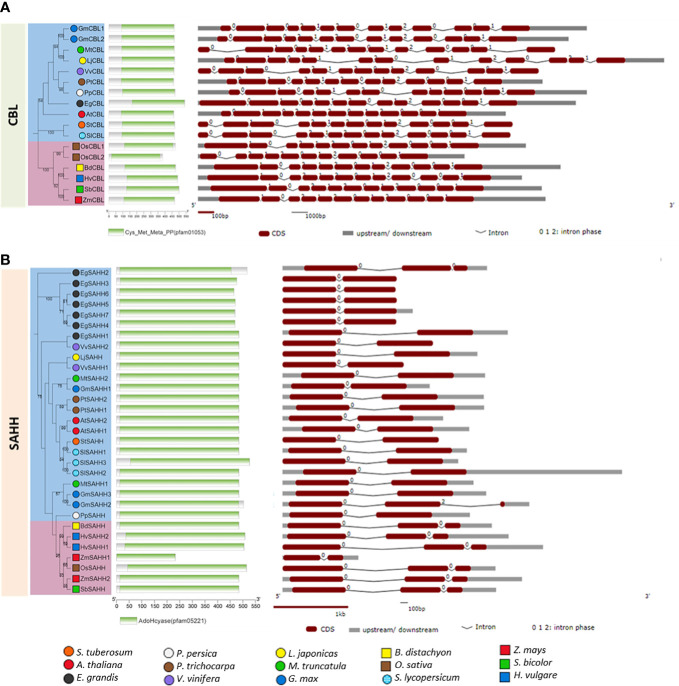
Phylogenetic relationship, conserved protein domains and gene structure of *CBL* **(A)** and *SAHH* **(B)** genes in various plant species. The phylogenetic trees were constructed *via* amino acid sequence alignment using ClustalW and the Neighbour-Joining method in MEGA-11, and evolutionary distances were computed using the JTT matrix-based method with the bootstrap test (1000 replicates). The tree was visualized on the iTOLv6 webtool (https://itol.embl.de/). The conserved protein domains were analyzed using the NCBI Batch CD-search webserver (https://www.ncbi.nlm.nih.gov/Structure/bwrpsb/bwrpsb.cgi, accessed on 25.10.2022) and were displayed by the TBtools software (Chen et al., 2018). The gene structure showing the intron/exon organization was generated by The Gene Structure Display Server (GSDS 2.0, Hu B. et al., 2015) tool (http://gsds.cbi.pku.edu.cn/, accessed on 24.10.2022). The dicot plants are presented in colored circles and the monocots are in colored squares. The *CBL* and *SAHH* genes from various plant species used here were as follows: AT3G57050 (*AtCBL*); Glyma.19G132000 (*GmCBL1*); Glyma.03G129700 (*GmCBL2*); Potri.016G038200 (*PtCBL*); PGSC0003DMG400029836 (*StCBL*); Solyc10g079720.1 (*SlCBL*); VIT_208s0007g05410 (*VvCBL*); Medtr1g064320 (*MtCBL*); Prupe.7G042400 (*PpCBL*); Eucgr.A02261 (*EgCBL*); Lj2g0003873 (*LjCBL*); Bradi1g47910 (*BdCBL*); HORVU7Hr1G028540 (*HvCBL*); Sobic.010G059200 (*SbCBL*); Zm00001d045153 (*ZmCBL*); LOC_Os06g07860 (*OsCBL1*); LOC_Os06g07960 (*OsCBL2*); AT4G13940 (*AtSAHH1*); AT3G23810 (*AtSAHH2*); Glyma.11G254700 (*GmSAHH1*); Glyma.05G152000 (*GmSAHH2*); Glyma.08G108800 (*GmSAHH3*); Potri.001G320500 (*PtSAHH1*); Potri.017G059400 (*PtSAHH2*); PGSC0003DMG400004572 (*StSAHH*);VIT_205s0029g00330 (*VvSAHH1*); VIT_217s0000g09840(*VvSAHH2*);Medtr8g083090 (*MtSAHH1*); Medtr3g084340 (*MtSAHH2*); Solyc09g092380.3 (*SlSAHH2*); Solyc09g092390.2 (*SlSAHH3*); Solyc12g098500.2 (*SlSAHH1*); Prupe.1G165200 (*PpSAHH*); Eucgr.D00281 (*EgSAHH1*); Eucgr.H02678 (*EgSAHH2*); Eucgr.H03173 (*EgSAHH3*); Eucgr.H03174 (*EgSAHH4*); Eucgr.H03176 (*EgSAHH5*); Eucgr.H03177 (*EgSAHH6*); Eucgr.H03180 (*EgSAHH7*); Lj6g0016083 (*LjSAHH*); LOC_Os11g26850 (*OsSAHH*); Zm00001eb090450 (*ZmSAHH1*); Zm00001eb170040 (*ZmSAHH2*); HORVU2Hr1G109370 (*HvSAHH1*); HORVU2Hr1G110120 (*HvSAHH2*); Sobic.005G112800 (*SbSAHH*) and Bradi4g19457 (*BdSAHH*).

Bioinformatics analyses allow us to supplement the experimental data and make new insights into the phylogenetic relationships and genomic/proteomic organization of genes involved in Hcy biosynthesis in plants. For the first time, we showed the *CBL* and *SAHH* genes from various plant species in terms of their phylogenetic relationships, protein domains and gene structure (exon/intron organization). We selected the model plants (Chang et al., 2016), including five monocots and ten dicots, and their sequences were obtained from PLAZA 5.0 and Phytozome databases (https://bioinformatics.psb.ugent.be/plaza/ and https://phytozome-next.jgi.doe.gov/, respectively, accessed on 22.10.2022). A total of 17 *CBL* and 31 *SAHH* genes were found to be distributed in 15 plant species (**Figure 2**). In the dicots, *G. max* had two *CBL* genes, while in the monocots *O. sativa* had two genes, while the other plants had only one CBL gene. For the *SAHH*, most of the dicots had 2-3 genes, except for *L. japonicas*, *P. persica* and *S. tuberosum*, which had one gene each. Notably, *E. grandis* contained seven *SAHH* members. In monocots, only *H. vulgare* had two *SAHHs*, while the rest of the analyzed plants contained one gene. Thus, gene duplication appears to have had a prominent role in the expansion of the *SAHH* family in dicot plants. Gene duplication, expansion, and eventual diversification are characteristics of the evolutionary process. The duplication of the *SAHH* genes might have contributed to evolving novel functions, such as growth and development, disease resistance, and stress tolerance (Panchy et al., 2016).

The phylogenetic analysis divided the plant *CBLs* (**Figure 2A**) and *SAHHs* (**Figure 2B**) into taxonomic groups, i.e. monocots and dicots. This is consistent with the divergent history of plant evolution (Chaw et al., 2004). The protein family domain analysis revealed that CBLs and SAHHs from all the plants contained the typical conserved domains, Cys_Met_Meta_PP (PF01053) and AdoHcyase (PF05221), respectively. Cys_Met_Meta_PP (Pyridoxal 5’-phosphate or PLP) is a versatile catalyst that acts as a coenzyme in a wide range of processes, including decarboxylation, deamination, and transamination. A number of pyridoxal-dependent enzymes involved in cysteine, homocysteine, and methionine metabolism have been reported (Ono et al., 1992; Barton et al., 1993). CBL belongs to the PLP-dependent enzyme fold-type I and shares considerable similarities with cystathionine γ- synthase. Both plant and bacterial CBLs are tetramers consisting of four identical subunits (Breitinger et al., 2001; Ravanel et al., 1996). Each active site has one cofactor molecule attached within a cleft produced between the dimer interfaces. Both monomer residues contribute to substrate binding and catalysis. AdoHcyase (adenosylhomocysteinase) is an enzyme of the activated methyl cycle that converts S-adenosyl-L-homocysteine into adenosine and homocysteine in a reversible manner (Turner et al., 2000; Yamada et al., 2005). The previous study suggested that in phylogenetically distinct land plants, SAHH forms oligomeric protein complexes, and the dominant protein complex is made up of a tetramer of the enzyme (Alegre et al., 2020). It was further shown that regulatory actions might be on the levels of protein complex formation and phosphorylation of this metabolically important enzyme.

The gene structure analysis revealed that the intron/exon arrangements in both monocots and dicots are similar (**Figure 2**). The size of the *CBL* genes ranged from 3 (*A. thaliana*) to 12 kbp (*L. japonicas*) in all the plants analyzed, with 12 introns in dicots and 10-11 introns in monocots. The *SAHH* gene size ranged from 1.5 (*E. grandis*) to 4.5 kbp (*S. lycopersicum*), with only one intron in dicots and mostly two introns in monocots. The *CBLs* of *V. vinifera* and *L. japonicas* had the large size of introns. This might be due to the abundance of repetitive/transposable elements (TEs) in their genomes, despite having genome sizes of only about 500 Mb (Jaillon et al., 2007; Li et al., 2020). Also, most of the *SAHHs* had one large intron, which might be due to the fact that the introns are generally relatively rich in repeats and TEs. The overall bioinformatics analysis revealed that a majority of plant *CBLs* and *SAHHs* are conserved in nature. Although monocots and dicots exhibit a few different features, they still exert a similar function.

Potentially present in both cytosol and chloroplast, methionine synthase (MS) methylates Hcy to methionine using a methyl group donor, 5-methyltetrahydrofolate (Hesse et al., 2004). In the model plant Arabidopsis the chloroplast AtMS3 is most likely required to methylate Hcy that is synthesized *de novo* in this organelle. In turn, the cytosolic isoforms AtMS1 and AtMS2 are engaged in the regeneration of Met from Hcy during the activated methyl cycle (Ravanel et al., 2004). Notably, a recent finding by Yan et al. (2019) revealed that *AtMS1* fulfills a regulatory role in transcriptional gene silencing. In addition to MS, cytosolic transfer of the methyl group to Hcy can be operated by Hcy *S*-methyltransferase (HcySMT) in the *S*-methyl-methionine (SMM) cycle (Ranocha et al., 2001). The SMM cycle also involves methionine *S*-methyltransferase engaged in the conversion of AdoMet to AdoHcy and Hcy to Met, respectively, linked with the conversion between Met and SMM (Sauter et al., 2013). It is worth noting that the maintenance of the AdoMet/AdoHcy ratio is considered a metabolic hallmark of the cellular methylation potential or methylation index (Hoffman et al., 1979; Moffatt and Weretilnyk, 2001; Kocsis et al., 2003). As indicated by Rudolf et al. (2021), AdoMet homeostasis, and thus balancing the AdoMet/AdoHcy ratio, depends on S-nitrosoglutathione reductase (GSNOR1). This enzyme controls the level of the S-nitrosoglutathione and S-nitrosation reactions in plant cells. Loss of GSNOR1 activity affects transmethylation reactions (Rudolf et al., 2021).

It was documented that an elevated level of Hcy is accompanied by decreased SAHH activity and over-accumulation of AdoHcy, which competitively inhibits AdoMet-dependent transmethylation, including DNA and histone methylation (Tehlivets et al., 2013). Thus, Hcy synthesis and AdoHcy removal by SAHH must be precisely and efficiently regulated. In Arabidopsis, SAHH1 and SAHH2 isoforms have been identified and the null mutation of SAHH1 results in embryonic lethality (Rocha et al., 2005). Moreover, an impaired SAHH1 function, including the knock-down *sahh1* and *homology-induced gene silencing 1* (*hog1*), resulted in delayed germination, growth, and morphological disorders (Rocha et al., 2005; Wu et al., 2009), indicating the significance of AdoHcy removal in plant cells. As it was stated by Alegre et al. (2020), SAHH1 functionality is crucial for plant metabolism at different developmental stages. Notably, SAHH activity can be regulated by NO, the key molecule in stress and developmental signaling. As documented, SAHH in pathogen-inoculated potato (Arasimowicz-Jelonek et al., 2016) and sunflower hypocotyls underwent nitration by peroxynitrite, resulting in the inhibition of SAHH activity (Chaki et al., 2009). Additional *in silico* analysis of the barley SAHH sequence showed that Tyr448 is the potential target for nitration (Chaki et al., 2009). According to Lozano-Juste et al. (2011), NO-dependent nitration may constitute an essential regulatory event that controls Met biosynthesis in plants. It was also proved that exogenous NO impairs MS activity (e.g., Danishpajooh et al., 2001), suggesting that MS nitration might result in reduced enzyme activity favoring elevated levels of Hcy in plant cells.

Although the trans-sulfuration reactions of Hcy to Cys are present in mammalian and fungal systems, the mechanism was not described in plants (Jakubowski and Guranowski, 2003).

## Hcy mode of action in plants

Although little attention has been paid to the research on the role of Hcy in plants, other non-proteinogenic amino acids such as ornithine, citrulline, arginosuccinate, homoserine, and cystathionine are well-recognized intermediates of plant metabolism (Jander et al., 2020). In plants, the non-proteinogenic amino acids show a broad range of roles, including anti-herbivory, antimicrobial, and allelochemical activity. Moreover, they are engaged in signaling, nitrogen storage, and general plant response to stresses (Bell, 2003). However, as underlined by Vranova et al. (2011), many aspects of the non-proteinogenic amino acids have been largely overlooked in plant research.

It is well established that Hcy is crucial for *de novo* methionine synthesis and AdoMet recycling, which constitutes a precursor of ethylene, polyamines, and nicotianamine. At the same time, it also controls DNA and histone methylation (Watanabe et al., 2021). Moreover, Hcy can manage *in vivo* serine biosynthesis *via* regulation of the 3-phosphoglycerate dehydrogenase (PGDH) activity. Okamura and Hirai (2017) showed that Arabidopsis AtPGDH1 and AtPGDH3 were activated under *in vitro* conditions by Hcy in a cooperative manner. The observed positive and tight regulation of AtPGDH1 and AtPGDH3 by Hcy may contribute to the balance between sulfur assimilation and tryptophan biosynthesis. Moreover, Hcy-mediated activation of the serine biosynthesis implicates the amino acid as a signaling molecule that enhances AdoMet production (Okamura and Hirai, 2017). Thus, the regulatory role of Hcy in plant cells should be assumed as an important intermediate in primary metabolism.

Besides the non-toxic effects of Hcy in plants, Jakubowski and Guranowski (2003) provided the first experimental line of evidence on the potentially toxic Hcy mode of action, as the formation of Hcy-thiolactone and Hcy-*N*-proteins was documented in plant cells (**Figure 1**). Inhibition of Hcy methylation to Met by the antifolate drug aminopterin in yellow lupine seedlings caused Hcy-*N*-proteins to become major metabolites of Hcy, next to Hcy-thiolactone. By deciphering the cellular metabolism of Hcy in living cells it was found that the conversion of Hcy to Hcy-thiolactone in plant cells is catalyzed by methionyl-tRNA synthetase (MetRS) in an error-editing reaction during protein biosynthesis when Hcy mistakenly replaces Met (Jakubowski, 2006). It was confirmed that Hcy editing to Hcy-thiolactone operates in organisms belonging to various domains of life. Analyses have shown that the Hcy-thiolactone pathway is predominant when reactions of remethylation or trans-sulfuration are affected by impaired enzymes regulating Hcy metabolism or by disorders in the supply of folate, vitamin B12, or vitamin B6 (Zimny et al., 2006). The reactive cyclic thioester of Hcy can be uniquely degraded in plant cells to Hcy by the constitutively expressed Hcy-thiolactone hydrolase, as documented in yellow lupine seedlings. Notably, the plant Hcy-thiolactone hydrolase is essentially different from the well-described human Hcy-thiolactonase/paraoxonase, as it does not require calcium for biological activity and can hydrolyze other sets of (thio)esters (Jakubowski and Guranowski, 2003).

Hcy-thiolactone was shown to provoke features of apoptosis in various types of cells such as placental trophoblasts, human endothelial and promyeloid HL-60 cells (Mercié et al., 2000; Huang et al., 2001; Kamudhamas et al., 2004). Promyeloid HL-60 cell treatment with Hcy significantly triggered intracellular hydrogen peroxide, which coincided with increased caspase 3 activity. Moreover, Hcy-mediated programmed cell death in human endothelial cells is followed by caspase 3 or a caspase-like protease activation (Zhang et al., 2001). Moreover, an excessive Hcy level upregulated the expression of autophagy-relevant proteins, such as Atg5, in human mesangial cells (Liang et al., 2019). Autophagy activation by amino acid starvation was found to promote Hcy-induced apoptosis in bovine aorta endothelial cells (Sato et al., 2020). Notably, overexpression of genes involved in Hcy biosynthesis, i.e., *CBL* and *SAHH*, was correlated in time with Hcy accumulation during hypersensitive cell death in potato immunity to *P. infestans*, suggesting an implication of Hcy in programmed cell death in plants (Arasimowicz-Jelonek et al., 2013).

## Hcy metabolism as a potential fingerprint of plant physiological disorders

Under normal conditions, the mean concentration of total Hcy (including disulfide-bound forms) in human plasma is ∼10 μmol/L. Thus, an elevated Hcy level has been related to inflammation processes and metabolism dysregulation leading to numerous cardiovascular and neurodegenerative disease states. The total pool of Hcy in lupine seedling hypocotyls was calculated at 4.3 μM (Jakubowski and Guranowski, 2003). In turn, the steady-state level of Hcy in leaves of the model plant Arabidopsis was assessed as < 1 pmol per mg fresh weight (Groth et al., 2016). However, a point mutation in the methylenetetrahydrofolate dehydrogenase/methenyltetrahydrofolate cyclohydrolase 1 (*MTHFD1*) gene of Arabidopsis provoked a disorder of folate metabolism, which caused the accumulation of Hcy in leaves to ∼7 pmol per mg fresh weight (Groth et al., 2016). Remarkably, sulfur starvation experiments revealed that the sulfur-deficient status in Arabidopsis seedlings did not affect the relative pool of Hcy (Nikiforova et al., 2005).

More recently, Watanabe et al. (2021) underlined that Hcy metabolism in plants can be altered under unfavorable environmental conditions. However, technical difficulties in its measurement contribute to a small number of data illustrating Hcy changes in plant cells. The Hcy accumulation and its localization were first documented in potato leaves inoculated with the causative agent of late blight using the immunohistochemical method (Arasimowicz-Jelonek et al., 2013). Interestingly, more pronounced immunofluorescence signals attributable to Hcy presence were observed primarily in healthy susceptible leaves rather than the resistant potato genotype. The Hcy-dependent signals were noticeable in the central vein and epidermal cells. The progressive development of late blight was correlated with over-accumulation of Hcy and upregulation of genes directly engaged in Hcy biosynthesis, *i.e.*, *CBL* and *SAHH* (Arasimowicz-Jelonek et al., 2013). On the other hand, susceptible potato leaf pretreatment with 100 µM Hcy and subsequent pathogen inoculation showed enhanced cytotoxicity, manifested by lipid peroxidation and rapid development of disease. The destructive effect was even more visible when leaves were treated with the antifolate drug - aminopterin, an inhibitor of Hcy methylation to Met. The set of experiments revealed that Hcy over-accumulation is engaged in a pathophysiological mechanism that abolishes basal resistance, suggesting Hcy monitoring as an informative hallmark characterizing plant susceptibility. The pathogen-induced Hcy accumulation was likely associated with the formation of Hcy metabolites, potentially favoring pathophysiological changes. It was earlier documented that an enhanced pool of S-nitrosothiols (SNOs) correlated with higher susceptibility to *P. infestans* (Floryszak-Wieczorek et al., 2012). As cellular SNOs include S-nitroso-Hcy, the metabolite could participate in SNO turnover controlling the expression of plant resistance to pathogenic microorganisms (Malik et al., 2011). It is worth noting that potato challenge with the potato virus Y at elevated temperatures also resulted in cultivar-dependent changes in the Hcy level (Fesenko et al., 2021; Spechenkova et al., 2021). However, upregulation of *CBL* correlated with an increased Hcy content was detected in the potato genotype displaying resistance to virus infection (Spechenkova et al., 2021). Moreover, in hypocotyls of yellow lupine seedlings growing under normal conditions Hcy-thiolactone accompanied the presence of Hcy at a concentration of < 0.6 μM and Hcy-N-protein <0.06 μM (Jakubowski and Guranowski, 2003). When the accumulation of Hcy was enhanced (to 245 μM) in response to aminopterin application, the formation of Hcy-thiolactone and Hcy-*N*-protein significantly increased in lupine hypocotyls, reaching 49.5 μM and 0.47 μM, respectively.

The AdoMet/AdoHcy ratio may also precisely reflect the plant’s physiological state and alterations in this proportion modify developmental and stress responses (Watanabe et al., 2021). Significantly, this quantitative relation may differ depending on external conditions. Experimentally established levels for AdoMet and AdoHcy in Arabidopsis were ∼15 and ∼0.5 pmol/mg fresh weight, respectively (Groth et al., 2016). Deprivation of mineral nutrients such as sulfur resulted in a significant drop in AdoMet in Arabidopsis seedlings, whereas AdoHcy remained unchanged and led to the diminished AdoMet/AdoHcy ratio (Nikiforova et al., 2005). Sulfur and iron deficiency also provoked perturbation of the AdoMet/AdoHcy ratio in Arabidopsis roots. Thus, stress conditions decreased the level of AdoHcy more than AdoMet, whereas the AdoMet/AdoHcy ratio increased (Forieri et al., 2017). The research has proven that the AdoMet amount is also crucial for the cell methylation status. In this regard, alterations in AdoMet-related metabolism affect the transcriptional status through epigenetic changes in histone modifications. Moreover, excessive Hcy is associated with the risk of downregulation of SAHH activity, including over-accumulation of AdoHcy, which suppresses AdoMet-dependent transmethylation, including DNA and histone methylation (Forieri et al., 2017). The potential toxicity of Hcy toward plant cells is summarized in **Figure 2**. Notably, plant-derived compounds such as resveratrol and curcumin have shown beneficial effects in reducing Hcy levels in clinical trials (Atazadegan et al., 2021). This seems to suggest that Hcy could be less destructive for plant cells, as they might be adapted to detoxify its excess by a broad range of mechanisms engaging various secondary metabolites. As reviewed by Atazadegan et al. (2021), the potential to diminish the Hcy level in animal cells has also been documented for black and green tea, cinnamon, garlic, and ginger extracts, as well as soybean.

## Conclusions

Our understanding of the Hcy metabolism and Hcy impact on animal and human pathophysiology has significantly advanced during the last decades. The current state of knowledge lets us see that Hcy is not only the immediate precursor of methionine, but can also provide an informative role on the plant physiological state. Recognizing Hcy as a standard marker of plant metabolic disorders caused by various stresses still seems rather far-fetched, so intensive research on the overall identification of Hcy derivatives and their potential biotoxic features should be a priority in future studies.

## Author contributions

JF-W, MA-J and ES-N wrote the manuscript. UT performed bioinformatic analysis and results interpretation. All authors contributed to the article and approved the submitted version.
